# Resistance, resilience, and recovery of salt marshes in the Florida Panhandle following Hurricane Michael

**DOI:** 10.1038/s41598-021-99779-8

**Published:** 2021-10-14

**Authors:** Katherine A. Castagno, Tori Tomiczek, Christine C. Shepard, Michael W. Beck, Alison A. Bowden, Kiera O’Donnell, Steven B. Scyphers

**Affiliations:** 1 Marine and Environmental Sciences, Northeastern University, Boston, MA 02115 USA; 2 The Nature Conservancy, Boston, MA 02111 USA; 3 Naval Architecture and Ocean Engineering, USA Naval Academy, Annapolis, MD 21402 USA; 4 The Nature Conservancy, Gulf of Mexico Program, Big Pine Key, FL 33043 USA; 5 Institute of Marine Sciences, University of California Santa Cruz, Santa Cruz, CA 95060 USA; 6 Present Address: Center for Coastal Studies, Provincetown, MA 02657 USA

**Keywords:** Natural hazards, Ecosystem services, Climate-change mitigation

## Abstract

Characterizing the fragility, resistance, and resilience of marshes is critical for understanding their role in reducing storm damages and for helping to manage the recovery of these natural defenses. This study uses high-resolution aerial imagery to quantify the impacts of Hurricane Michael, a category 5 hurricane, on coastal salt marshes in the Florida Panhandle, USA. Marsh damage was classified into several categories, including deposition of sediment or wrack, fallen trees, vegetation loss, and conversion to open water. The marshes were highly resistant to storm damages even under extreme conditions; only 2% of the 173,259 km^2^ of marshes in the study area were damaged—a failure rate much lower than that of artificial defenses. Marshes may be more resistant than resilient to storm impacts; damaged marshes were slow to recover, and only 16% of damaged marshes had recovered 6 months after landfall. Marsh management mattered for resistance and resilience; marshes on publicly-managed lands were less likely to be damaged and more likely to recover quickly from storm impacts than marshes on private land, emphasizing the need to incentivize marsh management on private lands. These results directly inform policy and practice for hazard mitigation, disaster recovery, adaptation, and conservation, particularly given the potential for more intense hurricane landfalls as the climate changes.

## Introduction

**Figure 1:**
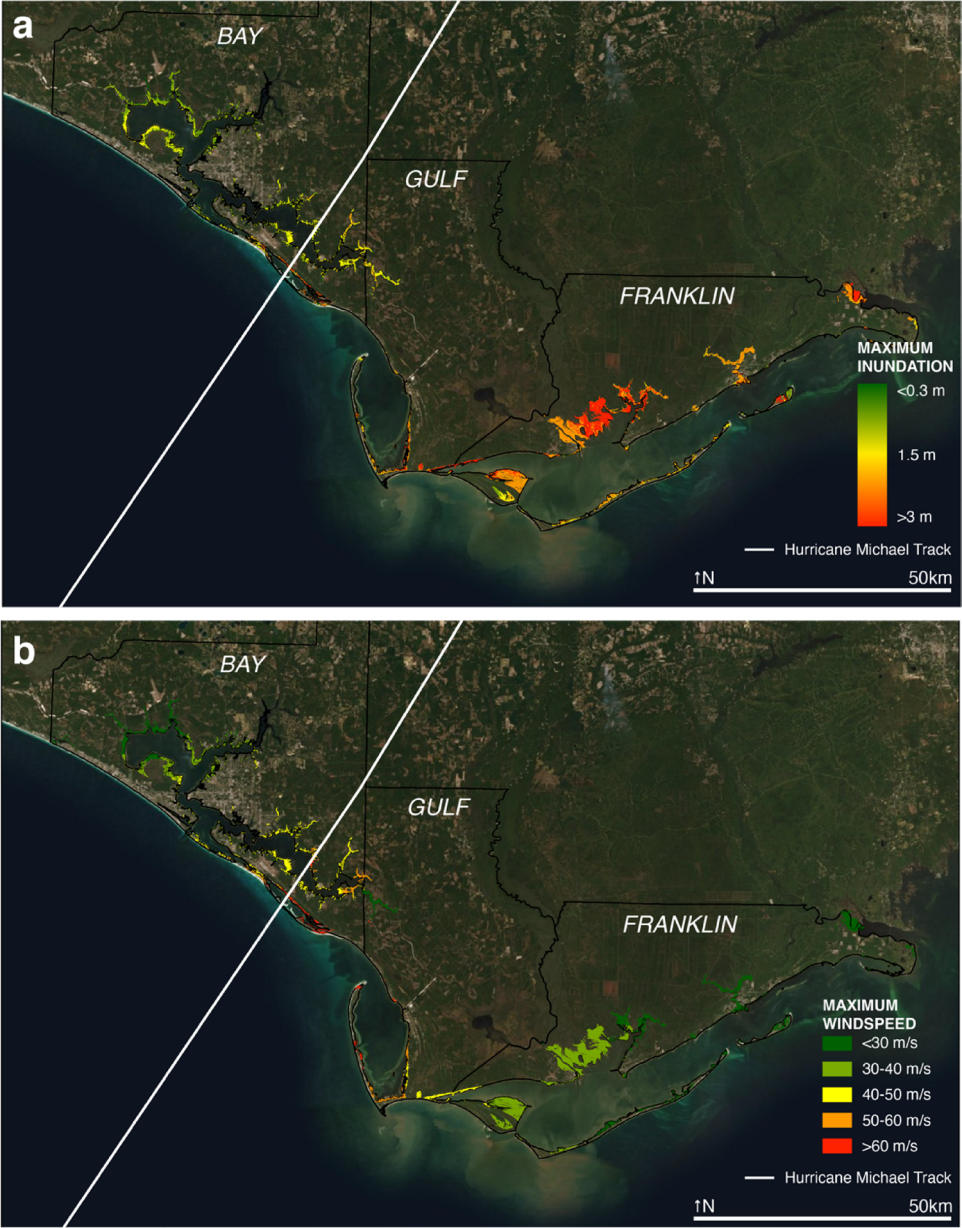
Hurricane Michael storm conditions and damage types. Aerial imagery for marshes in Bay, Gulf, and Franklin counties in the Florida Panhandle. Maps show (**a**) maximum inundation values, (**b**) maximum wind speed values. The track of Hurricane Michael is indicated by the white line. Map created with ESRI ArcGIS 10.8.0. Basemap sources: ESRI, Maxar, GeoEye, Earthstar Geographic, CNES/Airbus DS, USGS, AeroGrid, IGN.

Hurricane Michael made landfall in Mexico Beach, FL, on October 10, 2018, as a category 5 hurricane. As the first category 5 hurricane to make landfall in the contiguous United States since Hurricane Andrew in 1992, it brought devastating winds and storm surges to the Florida Panhandle, causing 16 deaths and $25 billion (2018 USD) damage in the United States^1^. Bay, Gulf, and Franklin counties in the Florida Panhandle contain more than 173,000 km^2^ of coastal salt marsh, largely dominated by *Juncus roemerianus* Scheele and *Sporobolus sp.* vegetation^2^. Salt marshes in Bay, Gulf, and Franklin counties experienced wind speeds greater than 60 m/s and inundations larger than 3 m (Fig. 1a,b).

Hurricanes and other intense storms impact marshes in a variety of different ways. Storms can be a major source of sediment deposition, which in turn increases marsh elevation and resilience to sea-level rise^3–8^. Wave action and inundation can also cause significant vegetation loss^9^, edge erosion^10,11^, and other marsh degradation, including pond formation^12^. Understanding how marshes are impacted by major storms is imperative to understanding system-wide coastal resistance and resilience.

Salt marshes have long been lauded for their coastal protection ecosystem services, in which marsh vegetation and elevation reduce storm wave heights and surge^13,14^. In 2012, coastal wetlands reduced inundation levels and thus prevented an estimated $625 million in direct flood damages from Hurricane Sandy in the northeastern United States^15^. In 2005, Hurricanes Katrina and Rita both caused devastating damage in southern Louisiana. Hurricane Rita was significantly less deadly and costly, despite making landfall at a similar size and intensity. This difference was largely due to travelling over 30–50 km of wetland before impacting a populated center, which accommodated extra surge more effectively than the large lagoons, highly-degraded wetlands, and artificial channels over which Hurricane Katrina traveled^16^.

An understanding of the resistance (or, inversely, “fragility” in risk industry models) and resilience of marshes is critical for understanding their role in reducing current and future storm damages and for identifying incentives for their conservation and resources for their recovery. To understand the consequences of storms for flooding, we must identify the conditions when defenses (natural and artificial) fail. Natural and nature-based features (NNBF), including salt marshes, are increasingly being leveraged for coastal protection to build resilience to storms and rising sea levels^17,18^. This understanding of how marshes are affected by and recover from major storm events can be incorporated into nature-based and hybrid shoreline stabilization and coastal protection designs. Building this knowledge base is particularly important as climate models suggest that hurricanes will increase in intensity as the climate continues to change^19^.

**Figure 2:**
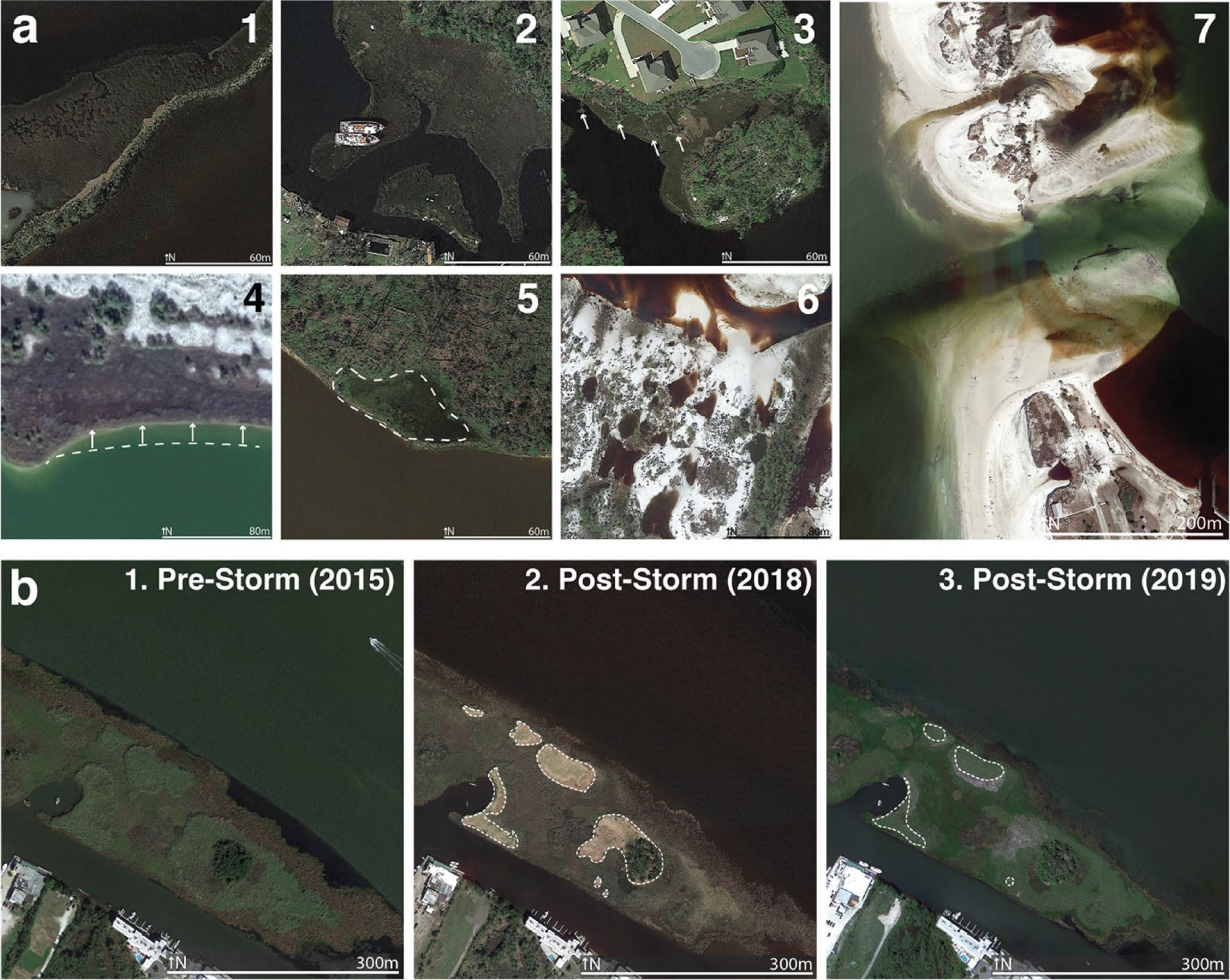
Examples of observed damage and recovery types. **(a)** Examples of marsh damage from Hurricane Michael include: (1) deposition of vegetation or sediment, (2) man-made debris, (3) fallen trees, (4) lateral erosion, (5) vegetation loss*, *(6) conversion to open water, and (7) channel cutting/widening. Example locations are indicated in Fig. S1. (**b)** Example of (1) pre-storm imagery (16 October 2015); (2) post-storm imagery (12 October 2018) with damaged areas indicated by dashed line; and (3) imagery 6 months after landfall (27 April 2019), with recovered areas indicated by dashed line. Example location indicated in Fig. 5, inset. Aerial imagery from Google Earth Pro 7.3.3.

This study uses analysis of high-resolution aerial imagery to quantify the impacts of Hurricane Michael on coastal salt marshes in Bay, Gulf, and Franklin counties in northwest Florida within the northern Gulf of Mexico of the continental United States (Fig. 2). Damage was determined visually using aerial imagery from Google Earth, which provided high- resolution aerial imagery from directly after Hurricane Michael’s landfall (11–12 October 2018) and prior to Hurricane Michael (October 2017, February 2017, October 2015). 30 m by 30 m marsh grid cells were classified into one of eight categories: no damage, deposition of vegetation or sediment, man-made debris, fallen trees, lateral erosion, vegetation loss*,* conversion to open water, or channel cutting/widening. In addition to quantifying damage, the damage classifications were related to storm and marsh characteristics to determine implications for marsh recovery, resistance, and resilience.

## Results

### Damage analysis

**Figure 3:**
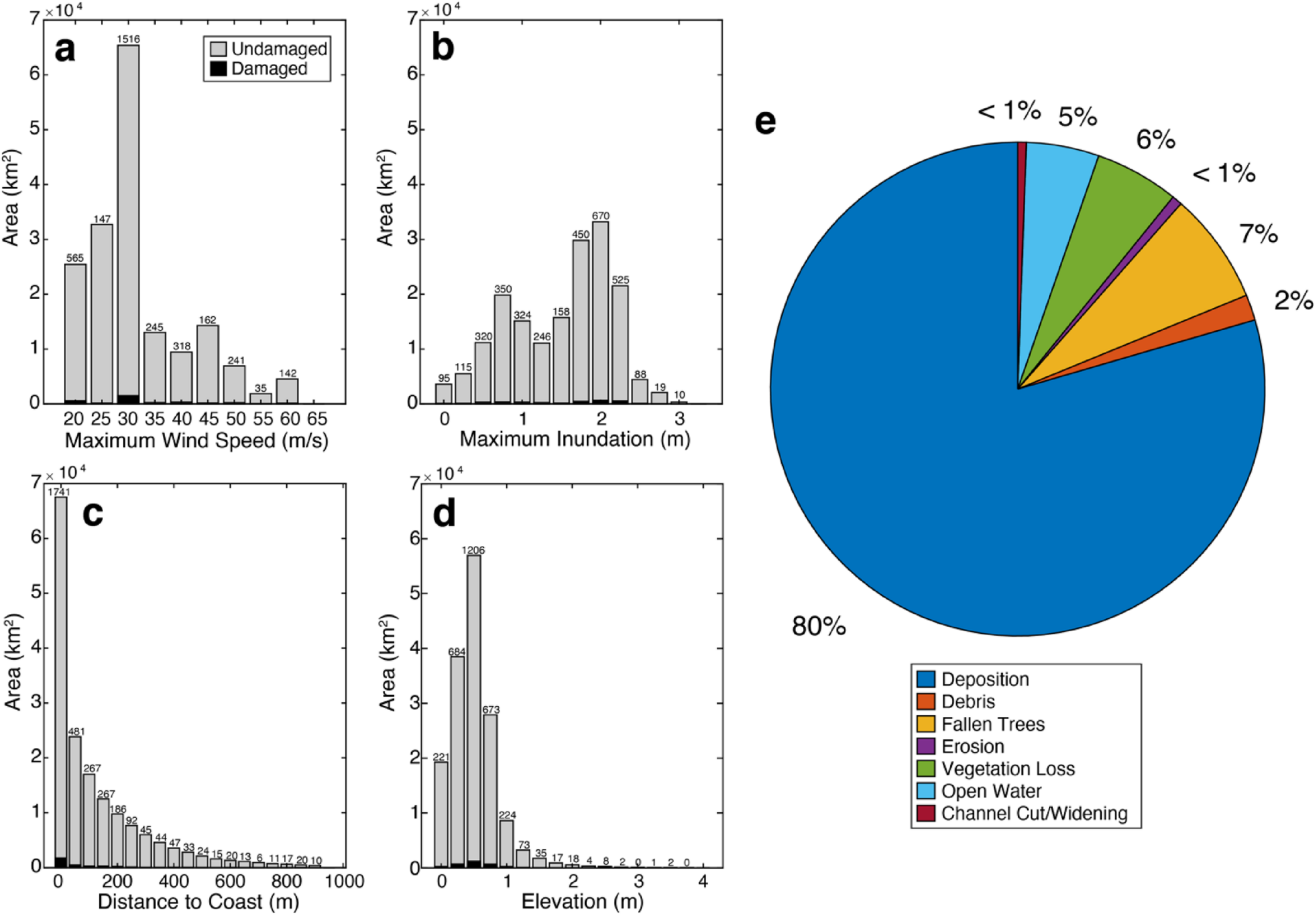
Marsh damage across all three study counties. Undamaged and damaged marsh area binned by (**a)** maximum windspeed (m/s; 5 m/s bin width), (**b)** maximum inundation (m; 0.25 m bin width), (**c)** distance to coast (m; 50 m bin width), and (**d)** elevation (m; 0.25 m bin width). (**e)** Pie chart of damage type across marshes in all three counties. Small numbers above each column indicate the number of square kilometers of damaged marsh.

**Figure 4:**
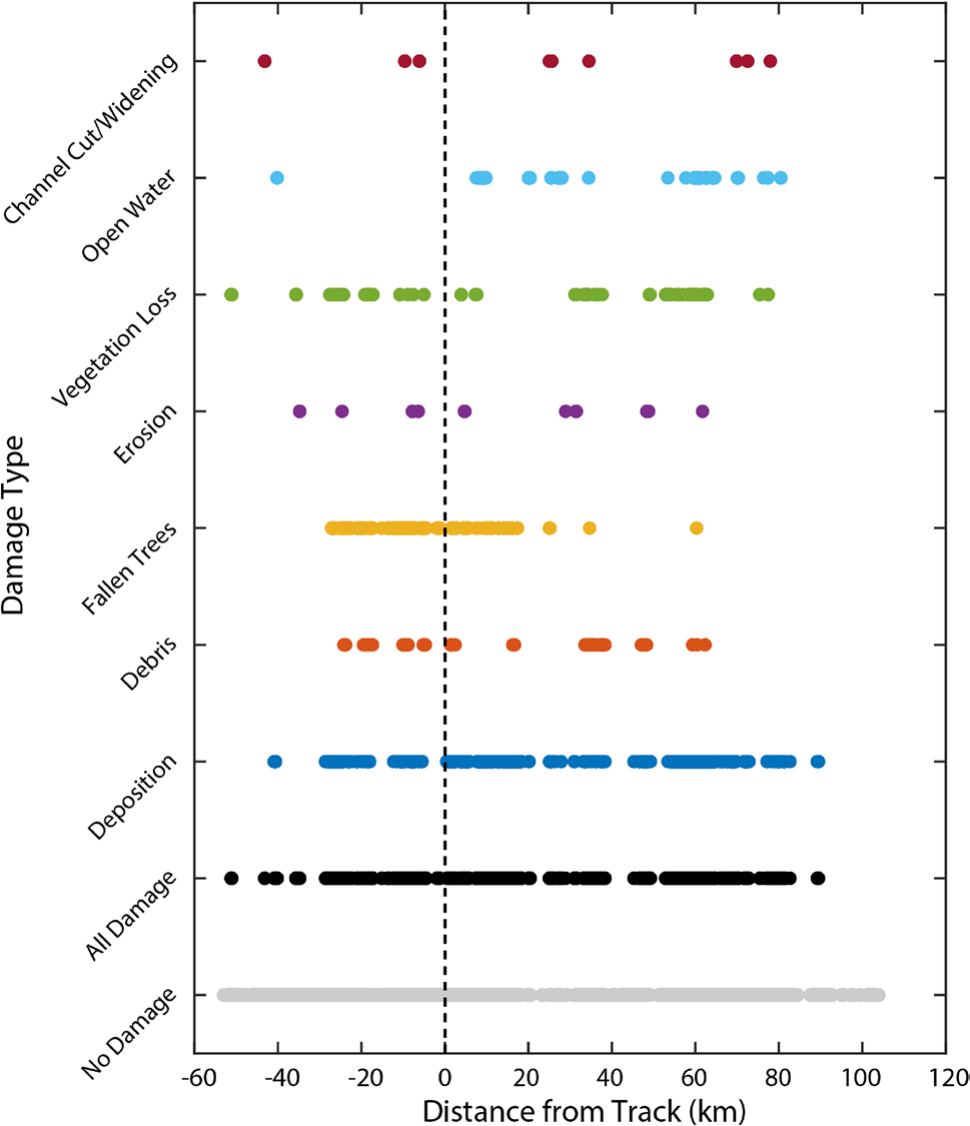
Marsh damage by damage type and distance from the track of Hurricane Michael. The track of the storm is indicated by the dashed line. Negative values indicate points to the west of the track; positive values indicate points to the east of the track.

Of the 173,259 km^2^ of marsh analyzed across Bay, Gulf, and Franklin counties, 3371 km^2^ were classified as damaged after Hurricane Michael (1.9% damaged; Fig. 3, Table 1). In Bay County, 1.8% of marshes were damaged. In Franklin County, 1.7% of marshes were damaged. In Gulf County, 4.3% of marshes were damaged, significantly more than that of the other two counties (*p* < 0.001). Across all three counties, 19% of damaged marsh occurred within 1 m of the coast, and 51.6% of damaged marsh occurred within 50 m of the coast (Table 2). There was no significant difference in distance from coast for damaged marsh across all study counties. Of total marsh damage, 85.8%, 87.9%, and 90.4% occurred at elevations less than 1 m in Bay, Franklin, and Gulf counties, respectively (Table 2). Marshes were more likely to be damaged east of the storm track (Fig. 4; *p* < 0.001). For marshes west of the storm track, 1.5% (666 km^2^) were damaged. 2.1% (2705 km^2^) of marshes east of the storm track were damaged.

**Table 1:** Area of marsh damage by county.

	Total marsh (km^2^)	Damaged marsh (km^2^)	Percentage damaged (%)
All counties	173,259	3371	1.9
Bay County	56,130	991	1.8
Gulf County	13,257	585	4.3
Franklin County	103,872	1795	1.7

**Table 2:** Storm and marsh property values for damaged marshes.

	Wind speed (m/s)	Inundation (m)	Distance to coast			Elevation	
	Mean ± std. (range)	Mean ± std. (range)	1 m (%)	50 m (%)	500 m (%)	0–0.5 m (%)	0–1 m (%)
All counties	36 ± 10 (22–65)	1.6 ± 0.7 (0–3.2)	19	51.6	95.1	32.8	88.5
Bay County	40.9 ± 12 (22–63)	1 ± 0.5 (0–3.2)	23.2	61.2	96.5	34.6	85.8
Gulf County	34.6 ± 17 (22–65)	1.2 ± 0.5 (0.1–3.2)	27.8	67.5	100	36.7	87.9
Franklin County	33.3 ± 3 (22–45)	2.1 ± 0.4 (0.2–2.9)	13.9	41.2	92.7	30.6	90.4

Damaged marshes were more likely to be on private land. Of all the salt marshes in this study, 76,929 km^2^ (44%) were on private land and 96,333 km^2^ (56%) were on public land. Of marshes that were damaged, 1633 km^2^ (48%) were on private land and 1738 km^2^ (52%) were on public land. The relationship between damage status and land ownership was significant (*X*^*2*^ [1, N = 192514] = 25.2, *p* < 0.001); damaged marshes were more likely to be on private land, given the difference in proportion of damaged to undamaged marsh on private land (2.1%) vs. public land (1.8%).

The majority of the marsh damage was due to deposition of vegetation or sediment (79.5% of damage in all counties; Table S1). Fallen trees composed 7.3% of damage across all counties, largely concentrated in Bay County (21.5% of damage), where Hurricane Michael made landfall, and decreasing with distance from the storm track. Vegetation loss and fragmentation composed 5.5% of damage across all counties, and conversion to open water composed 4.8% of damage across all counties (Table S1). The other damage categories (man-made debris on the marsh, lateral erosion, and channel cut/widening) composed less than 3% of damage across all counties.

**Figure 5:**
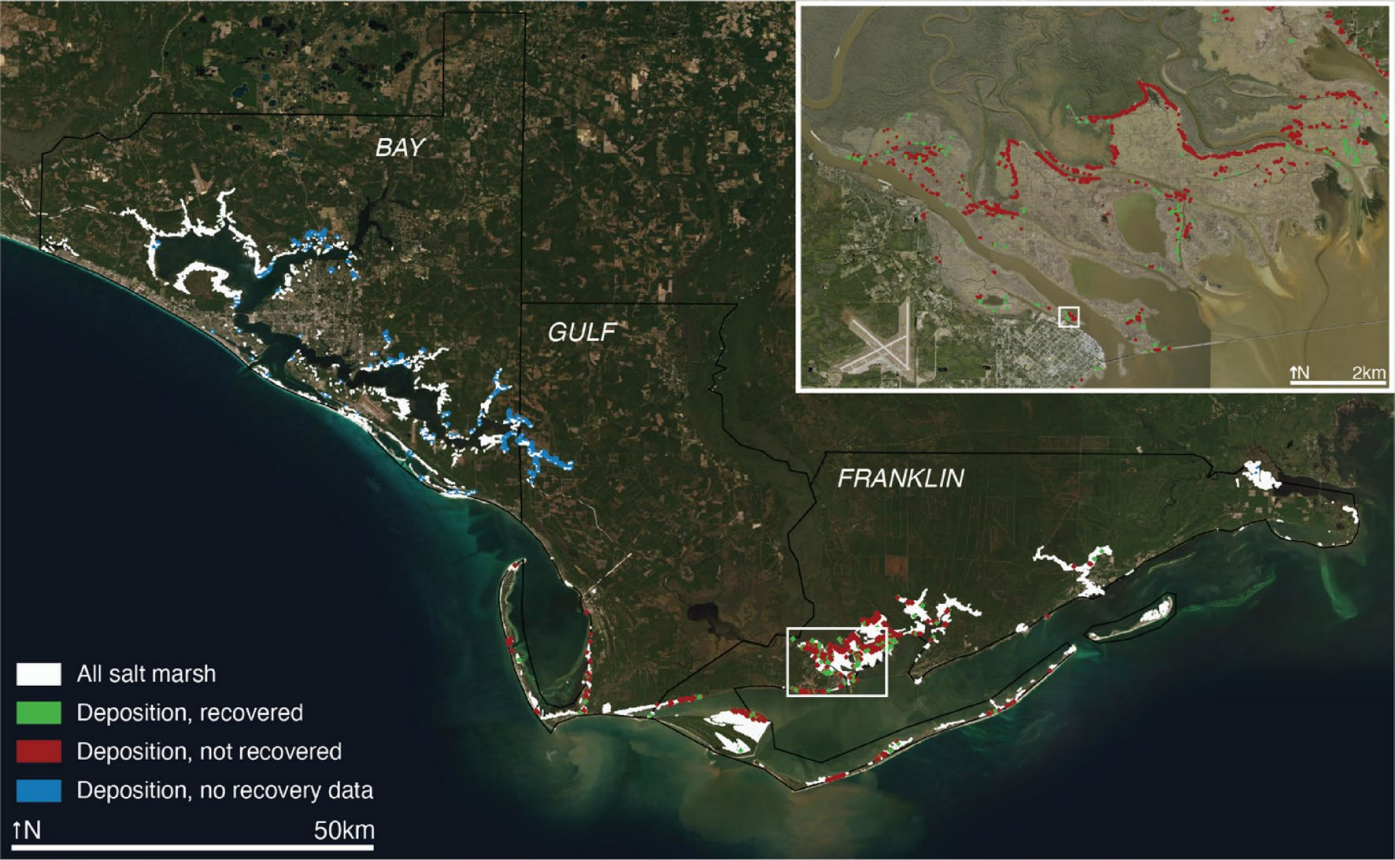
Map of deposition in study area. Areas of deposition of sediment or vegetation are indicated by green if recovered by April 2019, red if not recovered by April 2019, and blue if no recovery data. Grid cells with deposition are enlarged to show extent, not scale. Salt marsh area is indicated with white. Location of Fig. 2b indicated with white outline in inset map. Map created with QGIS version 3.18.3. Basemap sources: ESRI, Maxar, GeoEye, Earthstar Geographic, CNES/Airbus DS, USGS, AeroGrid, IGN.

Deposition (Fig. 5) occurred on marshes that were exposed to maximum wind speeds of up to 65 m/s (average 35 ± 9 m/s; Table S2) and experienced up to 3.2 m of inundation (average 1.6 ± 0.7 m). There was no significant difference in mean maximum wind speed across all three study counties (Fig. S2). Deposition-damaged marsh in Bay County experienced significantly lower average maximum inundation (0.9 ± 0.5 m/s) than that in Gulf County (1.1 ± 0.4 m/s), which was significantly lower than that in Franklin County (average 2.2 ± 0.3 m/s; *p* < 0.05). There was no significant difference in storm characteristics between undamaged marshes and damaged marshes experiencing deposition (Tables S2, S3). However, average wind speeds were statistically higher (*p* < 0.05) in marsh grid cells experiencing fallen trees and conversion to open water when compared to undamaged marsh cells (Table S2).

### Recovery analysis

**Figure 6:**
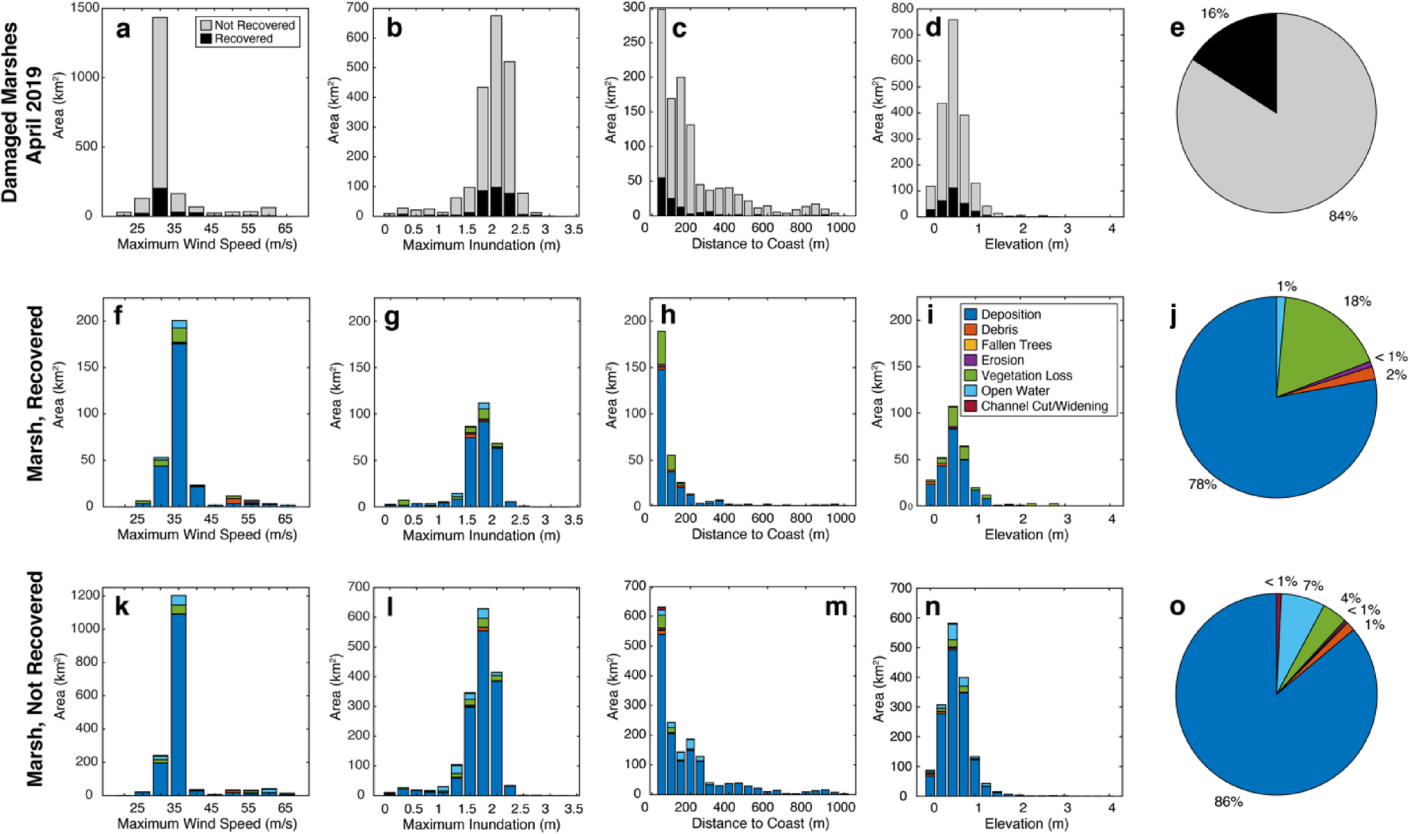
Analysis of recovery for damaged Panhandle marshes. Analysis is limited to marshes with aerial imagery from April 2019 (Fig. 2). (**a–d)** Recovered and non-recovered marsh area as a function of maximum wind speed, maximum inundation, distance to coast, and elevation, respectively. (**e)** Pie chart of ratio of recovered to non-recovered marshes. (**f–i)** Original damage type of recovered marsh area as a function of maximum wind speed, maximum inundation, distance to coast, and elevation, respectively. (**j)** Pie chart of distribution of original damage type of recovered marshes. (**k–n)** Damage type of non-recovered marsh area as a function of maximum wind speed, maximum inundation, distance to coast, and elevation, respectively. (**o)** Pie chart of distribution of damage type of non-recovered marshes.

Recovery from damage was analyzed for marshes in Gulf and Franklin counties (east of landfall) with aerial imagery from April 2019, six months after the landfall of Hurricane Michael (Fig. S2). Recovery was identified as an appearance in aerial photos similar to that of the pre-Michael imagery. In total, 1943.1 km^2^ of marsh (58% of damaged marsh across all three study counties) originally damaged from Hurricane Michael was included in the recovery analysis. As indicated in Table 3, of that area, 309.6 km^2^ (16%) recovered and 1633.5 km^2^ (84%) did not. Recovery was more likely for marshes damaged within 50 m of the coast (Fig. 6; Table S5). Inclusive of damage type, 21.8% of recovered marsh occurred within 1 m of the coast, and 61% of recovered marsh occurred within 50 m of the coast. Only 12.6% of non-recovered marsh occurred within 1 m of the coast, with 38.7% occurring within 50 m of the coast. Mean elevation for both recovered and non-recovered marshes was largely similar (0.6 ± 0.4 m and 0.6 ± 0.3 m, respectively; Table S6).

**Table 3:** Area of marsh recovery study and percent recovered by damage type.

	Recovered (km^2^)	Not recovered (km^2^)	Percent recovered by damage type (%)
All damage	309.6	1633.5	15.9
Deposition	241.2 (77.9%)	1406.7 (86.1%)	14.6
Debris	6.3 (2%)	23.4 (1.4%)	21.2
Fallen trees	0 (0%)	3.6 (0.2%)	0.0
Lateral erosion	2.7 (0.9%)	6.3 (0.4%)	30.0
Vegetation loss	54.9 (17.7%)	65.7 (4%)	45.5
Conversion to open water	4.5 (1.5%)	115.2 (7.1%)	3.8
Channel cut/widening	0 (0%)	12.6 (0.8%)	0.0

Marshes on public land were more likely to recover within 6 months. Of the 1943.1 km^2^ of salt marsh in the recovery study, 373.5 km^2^ was private land and 1569.6 km^2^ was public land. Of the private land, 92.7 km^2^ (14%) recovered and 280.8 km^2^ (86%) did not. Of the public land, 216.9 km^2^ (25%) recovered, and 1352.7 km^2^ (75%) did not. The relationship between recovery and land ownership was significant (*X*^*2*^ [1, N = 2159] = 30.3, *p* < 0.001).

Average mean maximum wind speeds were not significantly different between recovered and non-recovered groups (mean 35.1 ± 7 m/s) across all damage types and ranged from 22 to 65 m/s (Table S7). Average mean inundation was also not significantly different between recovered and non-recovered groups (2.0 ± 0.4 m) across all damage types and ranged from 0.2–3.2 m (Table S7).

Of the 1647.9 km^2^ of marsh with deposition damage, 14.6% had recovered by April 2019 (Fig. 6). For marshes that were damaged through deposition, the marshes that recovered experienced significantly lower (*p* < 0.05) maximum wind speeds (33.3 ± 5 m/s) than the marshes that did not recover (34.4 ± 5 m/s). Distributions were similar with marshes damaged from deposition: 26.1%, 61.2%, and 95.9% of recovered marshes were within 1, 50, and 500 m of the coast, respectively (Table S5). Of marshes that did not recover from deposition within 6 months, 12.8%, 38.3%, and 91.4% of non-recovered marshes were within 1, 50, and 500 m of the coast, respectively (Table S5). There was a significant difference in means between recovered and non-recovered marshes that experienced deposition (*p* < 0.05); recovered marshes were significantly closer to the coast. Marshes that recovered from deposition were at lower elevations (0.57 ± 0.3 m) than non-recovered marshes (0.64 ± 0.3 m).

## Discussion

Based on comparative analysis of aerial imagery, the marshes in the Florida Panhandle were overwhelmingly undamaged by Hurricane Michael. Of the 173,259 km^2^ of marsh analyzed across Bay, Gulf, and Franklin counties, only 1.9% (3371 km^2^) was classified as damaged after Hurricane Michael. Less than 2% of marshes were damaged in Bay and Franklin counties, and less than 5% of marshes were damaged in Gulf County. This result suggests that the majority of marshes in the study counties can withstand the effects of a category 5 hurricane and continue to provide coastal protection ecosystem services^29^. This also identifies marshes as more resistant than most other coastal defenses, including bulkheads. While this study did not examine bulkhead failure, similar studies found that 76% of bulkheads surveyed in North Carolina were damaged after Hurricane Irene^29^, and a large portion of bulkheads in the Florida Keys experienced significant damage after Hurricane Irma^31,32^.

Damage to marshes in the Florida Panhandle from Hurricane Michael was spatially distributed largely due to the storm track. Shortly before making landfall in Mexico Beach, the storm turned northeastward, influenced by the southern edge of mid-latitude westerlies^1^. The highest hurricane wind speeds are typically associated with the front-right quadrant of the storm and weaken rapidly after landfall, which was also true for Hurricane Michael^20^. As expected, the majority of damaged marshes (4.3%) were in Gulf County, which is directly southeast of landfall. Given the sizable decrease in wind speed and storm surge as the storm lost intensity over land, about half of the damage (51.6% across all counties) occurred within 50 m of the coast, and the vast majority of damage (95.1% across all counties) occurred within 500 m of the coast.

The types of damage were consistent with hurricane impacts in the Gulf of Mexico, with sediment deposition and wrack deposits among the most common morphological impacts from hurricane strikes^21,22^. The type of damage was also spatially distributed. Marshes in Bay County experienced the highest wind speeds and also experienced t he most damage from fallen trees, a largely wind-driven form of damage. Marshes in Franklin County experienced the largest inundations, as well as the most conversion to open water. Deposition of sediment or vegetation can be influenced by both wind and inundation, whether through aeolian transport^3–5,7,8,23^ or overwash^12^. Therefore, it is not surprising that deposition was both the most common as well as the most evenly spatially-distributed form of marsh damage. Vegetation loss, though less common than deposition, is also largely driven by high-velocity wind-driven currents; a particularly intense flow can pluck or denude a marsh of its vegetation^24,25^.

Marsh damage is also likely closely tied to physical properties of the marsh, in addition to the spatial component of storm conditions. The vast majority (88.5%) of marsh damage occurred at elevations of less than 1 m (NAVD88), consistent with both average marsh elevation and areas of greatest susceptibility to inundation. Localized susceptibility to damage may also indicate differences in marsh soil composition or shear strength^24,26^ or previous physical disturbance^27^. Given this study’s reliance on visual observations to determine damage, it is important to consider that not all storm-induced marsh damage can be ascertained visually; subsurface processes such as shallow subsidence or expansion can greatly influence the marsh’s elevation and resilience^14,28^.

It is also important to consider that, of the ~ 2% of marshes in the Florida Panhandle that were damaged, ~ 80% experienced deposition of sediment or vegetation on the marsh surface, which is a potentially less-permanent form of damage, especially compared to fallen trees or conversion to open water. Hurricanes regularly place thick sediment deposits on marshes in the Gulf of Mexico; short-term sedimentation rates in coastal Louisiana marshes after Hurricane Andrew increased for three months post-storm by 1–3 orders of magnitude compared to pre-storm rates, suggesting that resuspension and deposition continued well beyond the timeframe of the passing storm^33^_._ Deposition of sediment from storm overwash may actually benefit the system, increasing total marsh elevation and counteracting sea-level rise^8^. While storm-induced sedimentation may increase resilience, deposits that are too thick (> 5–10 cm^34^) may cause plant mortality and ultimately reduce the stability of the marsh and its resilience to storm impacts.

Much of the deposition on the marsh surface was rafted vegetation and wrack mats, which impacts marsh vegetation differently than sediment. This wrack deposition is consistent with past hurricane impacts in the Gulf of Mexico; wrack deposits from Hurricane Andrew completely buried vegetation in coastal Louisiana marshes, and areas of especially thick wrack were slow to recolonize, even a year after landfall^35^. A 1995 study, however, found that only 30% of wrack mats on a New England salt marsh damaged underlying vegetation^36^, and while wrack mats were a major cause of damaged low-marsh vegetation in a Virginia salt marsh, vegetation typically recovers^37^. This suggests that even the 2% of damaged marshes in the study area may be more resilient than originally thought.

Though the vast majority of marshes in the Florida Panhandle were not visually damaged by Hurricane Michael, of the damaged marshes with aerial imagery from six months after the storm, only 16% recovered. Marshes exposed to less extreme environmental conditions during the storm often were more likely to recover; this was the case for the relationship between mean maximum wind speed and deposition recovery, as well as maximum inundation and vegetation loss and conversion to open water.

Perhaps unsurprisingly, some forms of damage are easier to recover from than others. More than 45% of marshes experiencing vegetation loss had recovered within six months, whereas only 16% of marshes experiencing deposition of sediment or wrack, the most common form of damage, had visually recovered within 6 months. This is consistent with other studies; marshes in the Mississippi River Delta denuded by Hurricane Camile partially or completely recovered within one growing season, provided the root mat was not destroyed and the area was not permanently submerged^12^. This study is limited to 6 months after landfall, which is a relatively short time frame and does not include the spring and summer growing seasons. Given that other studies have shown that it can take over a year to recover marsh vegetation after disturbance (including storms and fire)^38^, and it is likely that more marsh recovery would be observed a full year after disturbance. Despite these considerations, this study provides important insight into the short-term recovery of vegetation (through recolonizing after plucking or growing through thin layers of deposition).

Once vegetation is substantially disturbed or submerged, however, recovery becomes more difficult, and it is these areas that might be prioritized for more active recovery interventions. Kirwan et al. (2008) found that disturbed areas experience decreased vertical accretion, which in turn causes localized submergence of the marsh platform, as well as potential expansion of channel networks, further destabilizing the marsh. Less than 4% of marsh that had been converted to open water and none of the marsh experiencing channel widening or cutting had recovered within 6 months. This is consistent with damage identified from a survey of hurricanes impacting southern Louisiana over the last 50 years^12^. Since newly formed or widening ponds or channels indicate significant loss of the marsh platform, it is far more difficult for the marsh to both recover elevation and regrow vegetation. Research has shown that ponds created by hurricane impacts could maintain their shape for decades and eventually become a permanent feature^39^.

This study found that though the vast majority of marshes in Bay, Gulf, and Franklin counties were not visually damaged during Hurricane Michael, of the 2% of marshes that did experience damage, 84% had not recovered within six months. This suggests that marshes may be largely resistant to storm impacts but not particularly resilient^19^, at least within the first six months of disturbance. Recovery rates for marshes on public land were significantly higher than private land. This speaks to the benefits of public land management and the need to create better incentives for marsh management on private lands. Additionally, the majority of marshes on public land are within larger wildlife refuges, state parks, or management areas and, as a result, may be less altered and more resilient to storm impacts.

Future damage surveys incorporating on-the-ground assessments and finer-resolution aerial imagery are recommended to fully understand the impacts of intense storms on salt marshes. Since this study, by design, only quantifies damage and recovery visible from aerial imagery, it is important to consider that there may be additional damage to the marshes that may be small, subtle, or belowground and therefore not observable at the scale of the imagery. While this study is specific to the inundation and hydrodynamic conditions associated with Hurricane Michael, the implications about marsh performance and recovery provide an important base for similar future assessments of future storm impacts.

Post-storm management actions may be key to increasing marsh resistance and resilience after storm impacts. As seen in this study, deposition is the one of the most common forms of marsh damage, but also one of the most easily reversible. While storm-induced sediment deposition is often largely beneficial to building marsh elevation^31^, wrack deposits may damage vegetation^36,37^, which in turn must recover by the next growing season to remain resilient to the next hurr icane season. Removing the thickest layers of wrack buildup to prevent vegetation death is a relatively straightforward management activity, which in turn will build the marsh’s resilience to future near-term storm impacts. Cost-effective restoration and recovery efforts should prioritize maintaining vegetation health, particularly in locations where the marsh platform is still intact post-storm. Post-storm wrack removal is a target for recovery funding that may be particularly beneficial.

Results of this study inform policy, funding, and approaches for hazard mitigation, disaster recovery, adaption, and conservation. Very few marshes were damaged or failed during a direct hit from one of the strongest hurricanes to impact the continental United States, and marsh failure rate was generally much lower than that seen for other artificial defenses, even in lower-intensity storms^30–32^. A portion of damaged marshes also began to recover within six months with few or no interventions, which artificial defenses cannot. Managed marshes on public lands are more likely to be both resistant and resilient to storm damage. Previous work has shown that marshes significantly reduced hurricane damage to property^15^ and are a cost-effective method of coastal protection^40^. Combined with our results, which indicate that marshes are highly resistant to storm impacts, these studies identify the need for additional public and private incentives, including insurance incentives and hazard mitigation funding, to conserve and restore marshes for their benefits to both people and property. After a storm, allocation of recovery funds to this natural, national infrastructure is imperative to improving management and recovery trajectories, particularly given the potential for more intense hurricane landfalls as the climate changes^19^.

## Methods

Salt marshes were identified in Bay, Gulf, and Franklin counties using the USFWS National Wetlands Inventory. Marshes were divided into 30 m by 30 m grids (0.9 km^2^). Areas of damage were determined visually using aerial imagery from Google Earth, which provided high-resolution aerial imagery from directly after Hurricane Michael’s landfall (11–12 October 2018). Imagery was compared to aerial imagery from prior to Hurricane Michael (October 2015). For parts of Gulf and Franklin county for which aerial imagery from April 2019 was available, marsh cells identified as damaged during Hurricane Michael were assessed for recovery (yes/no) after six months. Google Earth imagery sources are detailed in Table S8. Analysis was normalized to 200 m altitude and 0.9 km^2^ resolution. While damage assessment was performed by one person for the entire study area, multiple authors were involved in the damage determination methodology and agreed on criteria for damage in exemplar imagery. Randomized reanalysis indicates good agreement with the initial identification; repeating damage assessment for the majority of Bay County revealed no misclassified cells.

Each grid cell was classified as damaged or undamaged; undamaged marsh had no visual change from pre-storm imagery to post-storm imagery whereas marsh damage was identified as a visual change from pre-storm imagery to post-storm imagery. Damage was classified into one of seven categories. Deposition of vegetation or sediment was identified with wrack deposits or sand overwash deposits. Man-made debris included debris such as boats or damaged boardwalks on the marsh. Fallen trees were identified through visual identification. Lateral erosion was identified as a decrease in marsh platform between pre- and post-storm imagery. Vegetation loss was identified as areas of bare earth that were previously vegetated in pre-storm imagery. Conversion to open water was identified as areas of open water (typically small ponds) that were not present in pre-storm imagery. Channel cutting or widening were classified as areas where a thinner or no channel existed prior to the storm. Recovery was determined if the grid cell returned to pre-storm imagery in the imagery taken six months after landfall.

To determine factors correlated with and likely driving storm damages to marshes, damaged marsh cells were analyzed for various storm conditions, including maximum inundation (Coastal Emergency Risks Assessment; Table S9) and maximum windspeed (National Hurricane Center; Table S9). Marsh cells were also analyzed for properties including distance to coast, elevation, distance and orientation from the storm track, and land ownership. Distance to coast was measured from the closest edge of the cell linearly to the closest coastline using a coastline dataset from the Florida Fish and Wildlife Commission (Table S9). Grid elevation was determined using the National Elevation Dataset from USGS (Table S9). Grid cell distance from and orientation (east or west) to the storm track of Hurricane Michael was determined using NOAA Best Track data (Table S9). Land ownership, classified broadly as public or private, was classified using parcel data from the University of Florida GeoPlan Center (Table S9).

Differences between mean storm conditions by county and damage type were determined using one-way Analysis of Variance (ANOVA). Chi-squared tests were used to determine if there was a statistically significant difference between marsh damage and recovery on private and public lands.

## Supplementary Information

Supplementary Information.

## Data Availability

The geospatial data from this study is available at http://hdl.handle.net/2047/D20413409.
