# Supplementary material for: Resistance, resilience, and recovery of salt marshes in the Florida Panhandle following Hurricane Michael
Source: Sci Rep. 2021 Oct 14;11:20381. doi: 10.1038/s41598-021-99779-8 (PMC8516897; doi:10.1038/s41598-021-99779-8)
Supplement: Supplementary file 1 — Supplementary Information. [file 41598_2021_99779_MOESM1_ESM.pdf]

Resistance, resilience, and recovery of salt marshes in the Florida Panhandle following Hurricane Michael

Katherine A. Castagno[1,2,3], Tori Tomiczek[4], Christine C. Shepard[5], Michael W. Beck[6], Alison A. Bowden[2], Kiera O'Donnell[1], Steven B. Scyphers[1]

[1]Marine and Environmental Sciences, Northeastern University, Boston, MA 02115
[2]The Nature Conservancy, Boston, MA 02111
[3]Center for Coastal Studies, Provincetown, MA 02657, *current affiliation*
[4]Naval Architecture and Ocean Engineering, United States Naval Academy, Annapolis, MD 21402
[5]The Nature Conservancy, Gulf of Mexico Program, Big Pine Key, FL 33043
[6]Institute of Marine Sciences, University of California Santa Cruz, CA 95060

SUPPLEMENTAL INFORMATION

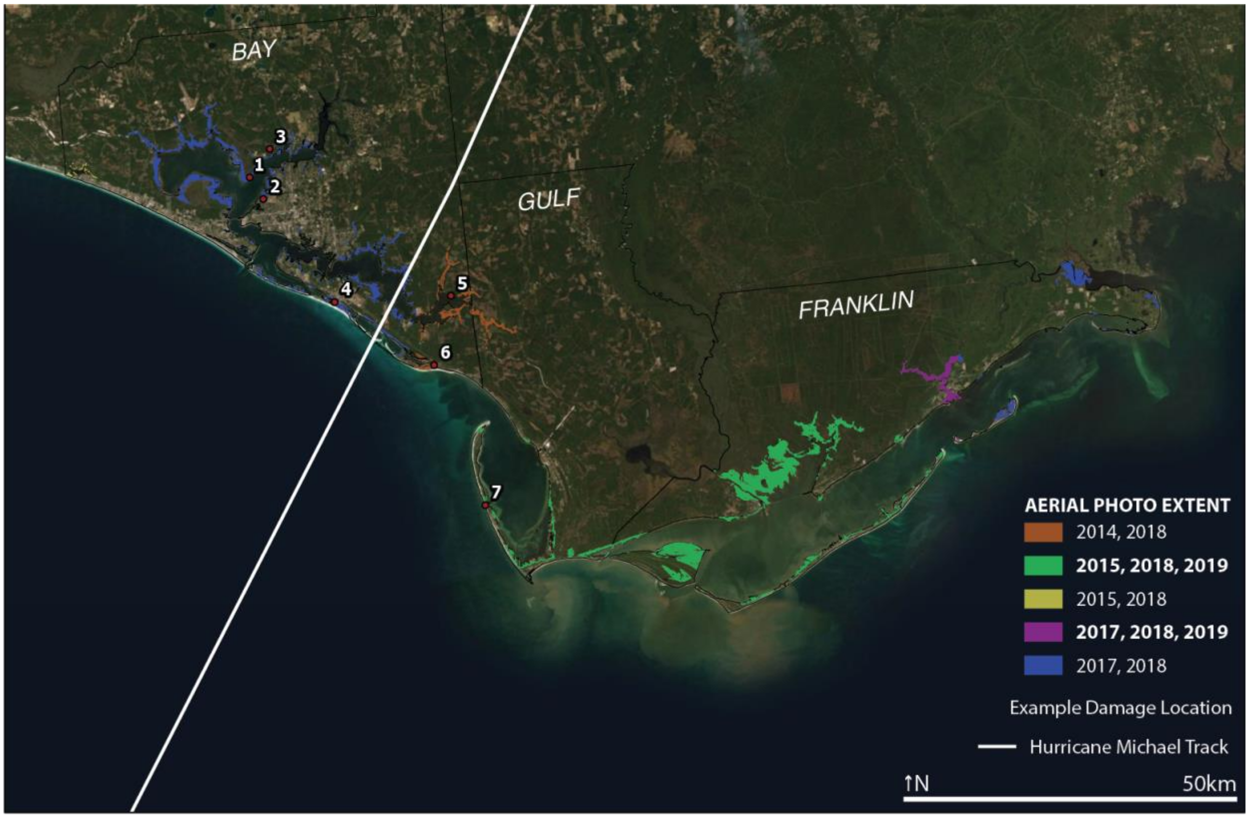

Figure S1. Extent of aerial imagery coverage. All marshes have aerial imagery from October 2018, within 1-2 days of Hurricane Michael's landfall. Areas with bolded dates (green and purple) have aerial imagery from April 2019, six months after landfall, and are used to study the marsh recovery from damage. Locations of aerial imagery from **Fig. 2a** are also indicated. Map created with ESRI ArcGIS 10.8.0. Basemap sources: ESRI, Maxar, GeoEye, Earthstar Geographic, CNES/Airbus DS, USGS, AeroGrid, IGN.

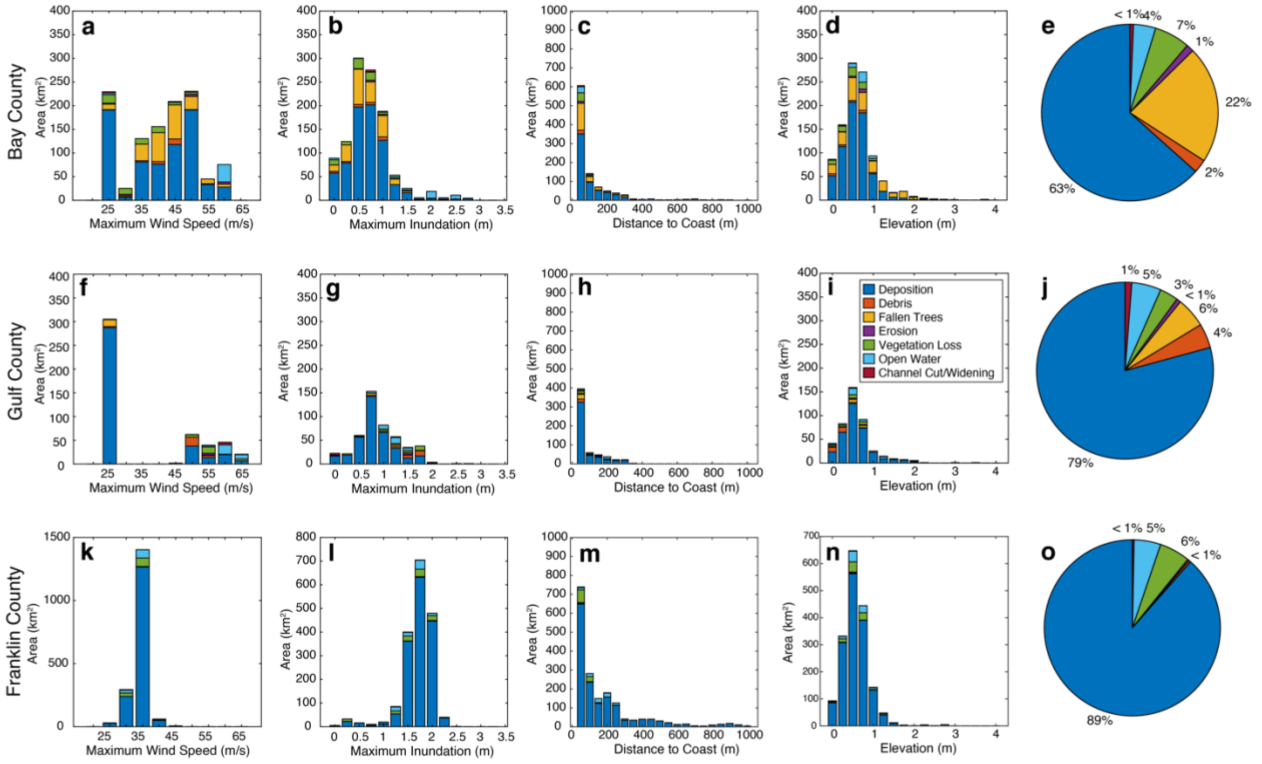

Figure S2. Analysis of damaged marshes by county. a-d) Distribution of marsh damage in Bay County by damage type as a function of maximum wind speed, maximum inundation, distance to coast, and elevation, respectively. **e)** Pie chart of marsh damage type distribution in Bay County. **f-i)** Distribution of marsh damage in Gulf County by damage type as a function of maximum wind speed, maximum inundation, distance to coast, and elevation, respectively. **j)** Pie chart of marsh damage type distribution in Gulf County. **k-n)** Distribution of marsh damage in Franklin County by damage type as a function of maximum wind speed, maximum inundation, distance to coast, and elevation, respectively. **o)** Pie chart of marsh damage type distribution in Franklin County.

Table S1. Area of marsh damage by damage type.

	All Counties	*Bay County*	*Gulf County*	*Franklin County*
All Damage, km^2 (%)	3,371 (100%)	991 (100%)	585 (100%)	1,795 (100%)
Deposition	2,680 (79.5%)	628 (63.4%)	464 (79.2%)	1,589 (88.5)
Debris	57 (1.7%)	24 (2.5%)	26 (4.5%)	6 (0.4%)
Fallen trees	247 (7.3%)	213 (21.5%)	32 (5.5%)	1 (0.1%)
Lateral erosion	23 (0.7%)	14 (1.4%)	5 (0.8%)	5 (0.3%)
Vegetation loss	185 (5.5%)	65 (6.5%)	20 (3.4%)	101 (5.6%)
Conversion to open water	160 (4.8%)	41 (4.1%)	32 (5.4%)	88 (4.9%)
Channel cut/widening	19 (0.6%)	6 (0.6%)	7 (1.2%)	5 (0.3%)

Table S2. Storm conditions for damaged marshes. Mean and standard deviation values of storm conditions that occurred on damaged marsh, separated by marsh condition. Parenthetical values indicate the range of values that damaged marshes experienced during Hurricane Michael. * indicates that the mean storm condition for damage type was statistically significantly different from the other study counties ($p<0.05$; one-way ANOVA). ** indicates that that the mean storm condition for damage type was statistically significantly higher than the storm conditions for undamaged marshes across all counties. *** indicates that that the mean storm condition for damage type was statistically significantly lower than the storm conditions for undamaged marshes across all counties.

Wind speed (m/s)	*All Counties*	*Bay County*	*Gulf County*	*Franklin County*
All damage *mean ± std. (range)*	36±10 (22-65)	40.9±12 (22-63)	34.6±17 (22-65)	33.3±3 (22-45)
Deposition	34.6±9 (22-65)	40±12 (22-63)	29.6±14 (22-65)	32.1±3 (22-34)
Debris	44.7±8 (22-56)**	42.2±6 (22-54)	48.5±9 (22-56)*	32.7±2 (25-34)
Fallen trees	41.4±9 (22-65)**	42.2±8 (22-60)	31.9±18 (22-65)	33±1 (31-34)
Lateral erosion	47.7±14 (22-63)**	46.3±16 (22-63)	58.6±1 (58-60)	33.5±0 (34-34)
Vegetation loss	35.4±9 (22-58)	33.2±9 (22-51)	52.8±7 (22-58)*	33±1 (31-34)
Conversion to open water	45.1±15 (25-65)**	59.2±11 (25-63)	62.3±3 (54-65)	32.4±1 (31-34)*
Channel cut/widening	44.5±16 (22-65)**	38±13 (25-49)	61.7±3 (54-65)*	31.9±1 (31-34)

Inundation (m)	*All Counties*	*Bay County*	*Gulf County*	*Franklin County*
All damage *mean ± std. (range)*	1.6±0.7 (0-3.2)	1±0.5 (0-3.2)	1.2±0.5 (0.1-3.2)	2.1±0.4 (0.2-2.9)
Deposition	1.6±0.7 (0.1-3.2)	0.9±0.5 (0.1-3.2)*	1.1±0.4 (0.1-3.2)	2.2±0.3 (0.3-2.9)*
Debris	1.4±0.7 (0.1-2.8)***	0.8±0.4 (0.1-1.4)*	1.8±0.4 (0.8-2.3)	2.3±0.2 (1.6-2.5)
Fallen trees	0.9±0.4 (0.1-2.3)***	0.9±0.4 (0.1-1.8)*	1.2±0.6 (0.3-2.3)	2.3±0.1 (2.1-2.4)
Lateral erosion	1.7±0.7 (0.2-2.7)	1.5±0.7 (0.7-2.7)	1.9±0 (1.8-1.9)	2.3±0.1 (2.3-2.4)
Vegetation loss	1.4±0.7 (0-2.5)***	0.7±0.4 (0-1.6)*	1.8±0.3 (1.2-2.1)	2.3±0.1 (1.9-2.5)
Conversion to open water	1.9±0.6 (0.2-3.2)**	2.2±0.8 (0.3-3.2)	1.4±0.3 (0.2-1.9)*	2.3±0.1 (2.1-2.4)
Channel cut/widening	1.1±0.7 (0.2-2.3)***	1±0.2 (0.7-1.2)	1±0.9 (0.3-2.3)	2.4±0 (2.4-2.4)

Table S3. Storm characteristics for damaged and undamaged marshes.

	**Damaged: Wind speed (m/s)**	**Undamaged: Wind speed (m/s)**	**Damaged: Inundation (m)**	**Undamaged: Inundation (m)**
	mean ± std. (range)	*mean ± std. (range)*	*mean ± std. (range)*	*mean ± std. (range)*
All Counties	36±10 (22-65)	34.4±9 (22-65)	1.6±0.7 (0-3.2)	1.6±0.7 (0-4.2)
Bay County	40.9±12 (22-63)	38.6±11 (22-63)	1±0.5 (0-3.2)	0.9±0.5 (0-4.2)
Gulf County	34.6±17 (22-65)	43.9±14 (22-65)	1.2±0.5 (0.1-3.2)	1.7±0.7 (0.1-3.7)
Franklin County	33.3±3 (22-45)	31±4 (22-45)	2.1±0.4 (0.2-2.9)	2±0.4 (0.1-3.6)

Table S4. Percentage of damaged grid cells within given values for specified marsh property.

	Distance to Coast			Elevation	
All Counties	1 m	50 m	500 m	0-0.5 m	0-1m
All damage	19.0	51.6	95.1	32.8	88.5
Deposition	18.9	49.4	94.3	33.5	90.4
Debris	28.6	68.3	100.0	66.7	93.7
Fallen trees	27.0	68.2	98.2	28.5	69.0
Lateral erosion	60.0	84.0	100.0	28.0	92.0
Vegetation loss	11.7	67.5	95.1	28.6	86.9
Conversion to open water	5.6	30.9	100.0	18.0	88.8
Channel cut/widening	47.6	85.7	100.0	52.4	76.2
Bay					
All damage	23.2	61.2	96.5	34.6	85.8
Deposition	21.4	55.8	97.1	36.3	90.7
Debris	33.3	81.5	100.0	55.6	88.9
Fallen trees	25.8	67.1	98.0	28.6	68.3
Lateral erosion	60.0	73.3	100.0	26.7	93.3
Vegetation loss	19.4	70.8	86.1	41.7	87.5
Conversion to open water	13.3	77.8	100.0	13.3	91.1
Channel cut/widening	57.1	100.0	100.0	57.1	71.4

	Distance to Coast			Elevation	
Gulf					
All damage	27.8	67.5	100.0	36.7	87.9
Deposition	31.6	74.0	100.0	35.0	88.0
Debris	27.6	62.1	100.0	89.2	100.0
Fallen trees	38.1	81.0	100.0	23.8	76.2
Lateral erosion	100.0	100.0	100.0	40.0	100.0
Vegetation loss	4.5	59.1	100.0	31.8	100.0
Conversion to open water	5.7	20.0	100.0	22.9	77.1
Channel cut/widening	50.0	87.5	100.0	50.0	75.0
Franklin					
All damage	13.9	41.2	92.7	30.6	90.4
Deposition	14.8	40.8	91.7	32.0	90.8
Debris	14.3	42.9	100.0	28.6	85.7
Fallen trees	100.0	100.0	100.0	100.0	100.0
Lateral erosion	20.0	100.0	100.0	20.0	80.0
Vegetation loss	8.0	67.0	100.0	19.6	83.9
Conversion to open water	2.0	13.3	100.0	18.4	91.8
Channel cut/widening	33.3	66.7	100.0	50.0	83.3

Table S5. Marsh recovery and distance to coast. Percentage of marsh damage in the recovery study within 1 m, 50 m, and 500 m of the coast, respectively. Mean and standard deviation values of marsh distance from coast in the recovery study, separated by marsh condition. Parenthetical values indicate the range of distance from coast in the recovery study. Bold numbers indicate that the mean distance from coast for damage type was statistically significantly different between recovered and non-recovered marshes (p<0.05; one-way ANOVA).

	**Distance to Coast (%)**			
Recovered	*1 m*	*50 m*	*500 m*	*mean ± standard deviation (range)*
All damage	21.8	61.0	96.8	88±186 (0-1807)
Deposition	26.1	61.2	95.9	**97±208 (0-1807)**
Debris	0	42.9	100.0	73±53 (10-142)
Fallen trees	none	none	none	none
Lateral erosion	100.0	100.0	100.0	0
Vegetation loss	3.3	65.6	100.0	44±27 (0-124)
Conversion to open water	0.0	0.0	100.0	242±76 (131-319)
Channel cut/widening	none	none	none	none

Not Recovered	*1 m*	*50 m*	*500 m*	*mean ± standard deviation (range)*
All damage	12.6	38.7	92.6	164±217 (0-1276)
Deposition	12.8	38.3	91.4	**175±230 (0-1276)**
Debris	23.1	57.7	100.0	69±759 (0-207)
Fallen trees	25.0	50.0	100.0	85±87 (0-179)
Lateral erosion	42.9	100.0	100.0	13±18 (0-44)
Vegetation loss	11.0	65.8	100.0	50±58 (0-407)
Conversion to open water	3.1	15.6	100.0	141±76 (0-302)
Channel cut/widening	42.9	78.6	100.0	39±69 (0-185)

Table S6. Marsh recovery and elevation. Mean and standard deviation values of elevation of marshes in the recovery study, separated by marsh condition. Parenthetical values indicate the range of elevations of marshes in the recovery study. Bold numbers indicate that the mean elevation for damage type was statistically significantly different between recovered and non-recovered marshes ($p<0.05$; one-way ANOVA).

Elevation (m)	***Recovered***	***Not Recovered***
All damage<br>*mean ± standard deviation (range)*	0.6±0.4 (0-2.6)	0.6±0.3 (0-4.2)
Deposition	**0.57±0.3 (0-1.8)**	**0.64±0.3 (0-4.2)**
Debris	0.3±0.2 (0.1-0.6)	0.4±0.3 (0-1.5)
Fallen trees	none	0.7±0.5 (0-1.1)
Lateral erosion	0.6±0.2 (0.5-0.8)	0.8±0.8 (0.3-2.5)
Vegetation loss	**0.9±0.6 (0-2.6)**	**0.7±0.4 (0-2.7)**
Conversion to open water	**1.1±0.7 (0.3-2)**	**0.7±0.3 (0-1.5)**
Channel cut/widening	none	0.5±0.7 (0-2.1)

Table S7. Marsh recovery and storm condition. Mean and standard deviation values of storm conditions that occurred on marshes in the recovery study, separated by marsh condition. Parenthetical values indicate the range of values that marshes in the recovery study experienced during Hurricane Michael. Bold numbers indicate that the mean storm condition for damage type was statistically significantly different between recovered and non-recovered marshes (p<0.05; one-way ANOVA).

Wind speed (m/s)	***Recovered***	***Not Recovered***
All damage<br>*mean ± standard deviation (range)*	35.1±7 (22-65)	35.1±7 (22-65)
Deposition	**33.3±5 (22-60)**	**34.4±5 (22-65)**
Debris	49.8±3 (42-51)	48.5±7 (34-56)
Fallen trees	none	54.2±15 (34-65)
Lateral erosion	**58.9±1 (58-60)**	**45.7±9 (34-58)**
Vegetation loss	35.1±8 (22-58)	37.8±8 (22-56)
Conversion to open water	50.5±20 (29-65)	40±13 (29-65)
Channel cut/widening	none	47.7±17 (22-65)

Inundation (m)	***Recovered***	***Not Recovered***
All damage<br>*mean ± standard deviation (range)*	2±0.4 (0.2-3.2)	2±0.4 (0.2-3.2)
Deposition	2.1±0.3 (0.2-2.6)	2.1±0.4 (0.2-3.2)
Debris	2.2±0.3 (1.7-2.8)	1.9±0.4 (0.8-2.6)
Fallen trees	none	2.2±0.2 (2-2.3)
Lateral erosion	1.8±0 (1.8-1.9)	2±0.8 (0.2-2.6)
Vegetation loss	**1.7±0.6 (0.2-2.4)**	**1.9±0.5 (0.2-2.5)**
Conversion to open water	**1.3±0.3 (0.7-1.6)**	**1.8±0.4 (0.2-2.5)**
Channel cut/widening	none	1.2±0.8 (0.2-2.3)

Table S8. Dates and sources of aerial imagery capture for each county. Google Earth imagery analysis was normalized to 200 m altitude and 0.9 km^2 resolution.

County	*Image Type*	*Date*	*Source*	*Resolution*	*Access*
Bay	Pre-Storm	16 October 2015	USDA NAIP	1-m GSD natural-color orthoimagery	https://earthexplorer.usgs.gov/
	Post-Storm	11 October 2018	2018 NOAA NGS Emergency Response Imagery: Hurricane Michael	25-cm GSD natural-color imagery	https://www.fisheries.noaa.gov/inport/item/57759
	Recovery	*no imagery*	*n/a*	*n/a*	*n/a*
Gulf	Pre-Storm	16 October 2015	USDA NAIP	1-m GSD natural-color orthoimagery	https://earthexplorer.usgs.gov/
	Post-Storm	12 October 2018	2018 NOAA NGS Emergency Response Imagery: Hurricane Michael	25-cm GSD natural-color imagery	https://www.fisheries.noaa.gov/inport/item/57759
	Recovery	27 April 2019	Florida Department of Transportation	0.5-ft GSD natural-color orthoimagery	https://www.fdot.gov/gis/aerialmain.shtm
Franklin	Pre-Storm	16 October 2015	USDA NAIP	1-m GSD natural-color orthoimagery	https://earthexplorer.usgs.gov/
	Post-Storm	11 October 2018	2018 NOAA NGS Emergency Response Imagery: Hurricane Michael	25-cm GSD natural-color imagery	https://www.fisheries.noaa.gov/inport/item/57759
	Recovery	27 April 2019	Florida Department of Transportation	0.5-ft GSD natural-color orthoimagery	https://www.fdot.gov/gis/aerialmain.shtm

Figure S9. Sources for datasets used in analysis.

Dataset	*Source*	*Date Published*	*Date Accessed*	*Access*
Aerial imagery	*see Table S8*	October 2015-April 2019	May 2020	Google Earth Pro
Salt marsh boundaries	USFWS National Wetlands Inventory	May 2020	May 2020	https://www.fws.gov/wetlands/
Hurricane Michael maximum inundation	Coastal Emergency Risks Assessment	December 2019	May 2020	https://cera.coastalrisk.live/
Hurricane Michael maximum wind speed	National Hurricane Center	May 2019	May 2020	https://www.nhc.noaa.gov/data/tcr/
Hurricane Michael storm track	National Hurricane Center	May 2019	May 2020	https://www.nhc.noaa.gov/data/tcr/
Florida shoreline	Florida Fish and Wildlife Commission	February 2002	May 2020	https://geodata.myfwc.com/datasets/florida-shoreline-1-to-40000-scale/
Elevation	USGS National Elevation Dataset	March 2020	July 2020	https://ned.usgs.gov/
Florida statewide parcel data	University of Florida GeoPlan Center	March 2018	July 2020	https://download.fgdl.org/pub/state/parcels_2017.zip
